# An Account of the Rise, Progress, and Present State of the Pennsylvania Hospital; the Pride of Philadelphia

**Published:** 1803-03-01

**Authors:** 


### *An* ACCOUNT *of the* RISE, PROGRESS, *and* PRESENT STATE *of the* PENNSYLVANIA HOSPITAL; *the Pride of Philadelphia.*

THIS humane and benevolent Institution was founded by the Contributors, in the year 1752, for the relief of the lunatics and sick poor of Pennsylvania, and has been supported by them ever since, *with legacies and private contributions.*

By a late act of Assembly, the contributors have liberty to graft upon it, a Lying-in and Foundling Hospital, as soon as money sufficient to carry it on can be raised.

They consist of persons who have given ten pounds, or more, and are incorporated by the name and title of, *Contributors to the Pennsylvania Hospital;* such may vote at elections for managers, or be voted for, but derive no personal interest from the act of incorporation. A sum, less than ten pounds, is called a donation.

The contributors have perpetual succession, with power to elect twelve managers, a treasurer, and all other officers of the institution, and make RULES for well-ordering the house. They may receive and take the lands, hereditaments, and tenements, not exceeding the yearly value of one thousand pounds, of the gift, alienation, bequest or devise of any person or persons whomsoever, and of any goods and chattels whatsoever; provided, that *no general meeting of the contributors, or persons acting under them, shall employ any money or other estate, expressly given to the capital stock of the Hospital, in any other way than by applying its annual interest or rent towards the entertainment and care of the sick and distempered poor, that shall from time to time be brought and placed therein, for the cure of their diseases, from any part of the state, without partiality or preference.*

If there should not be a constant succession of contributors to meet yearly and chuse managers, then the hospital, its estate and affairs, and all the management thereof, are to be under the direction of such persons as the legislature may appoint.

By a law of the contributors, the power of directing the manner and terms of receiving and discharging patients, is transferred to the managers, who made a rule, if there should be room in the Hospital, (after as many poor patients are accommodated as the interest of the capital stock can support) to take in such others as they can on reasonable rates rates agree for; and that the profits arising from boarding and nursing such patients, shall be appropriated to the same uses as the interest money of the public Stock.—The price of board is various, according to the applicants ability to pay, and changes with the rise and fall of provisions, &c.

The Overseers of the Poor of Pennsylvania, and religious societies therein, who support the poor by their own voluntary subscriptions, pay but *three dollars* a week, which is about the first cost of one person's maintenance, including medicine and all charges, except cloathing and funeral expences.

The overseers of the poor of other States pay *four dollars;* private patients, who are residents of Pennsylvania, from *three and a half to six dollars;* and non-residents from *four and a half to eight dollars.*—Every patient may chuse his own physician, but he must be one of the house physicians.

An amputation of a limb is not to be performed, unless the patient consents to it; nor then, unless three physicians agree to it, after a consultation on the case.

The sick, especially the stranger, finds it his interest to prefer the Hospital to any tavern, or boarding-house, for many reasons—

*First,* Because the physicians are the most eminent.

*Secondly,* The nurses are the most experienced.

*Thirdly,* The apartments are the most convenient.

*Fourthly,* The price of board is lower than any individual can take.—And,

*Lastly,* The patient has the satisfaction to know, if there is any profit it is given to the poor.

A physician of the Hospital must be twenty-seven years of age before he can be elected; and serve the poor gratis.

The duty of the resident apothecary is to attend the library, exhibit and explain the museum, administer prescriptions, and visit and dress the patients.

Two managers and physicians meet every fourth and seventh day in the Hospital (being market days) at eleven o'clock of the forenoon, to admit and discharge patients.

At intervening times, the applicant must repair to one of the monthly physicians, who, if he considers the case a proper one, will certify it in writing to the sitting Manager, and he will take the usual security, and give an order for admission.

Overseers of the poor from the country, who bring a patient, must have a certificate signed by the magistrates, denoting that they are in office, and the pauper proposed for admission resides in their district, or their application will be rejected.

Persons with infectious diseases are not to be received, nor incurable cases, lunatics excepted—but, any person living in or near Philadelphia, receiving by accident a desperate wound, or having a fractured limb, just broke, may be brought to the Hospital without an order, and he will be received day or night, *provided* he is brought in immediately.

From the time the hospital was founded there have been admitted into it about ten thousand patients, great numbers of whom have been lunatics; some of these have been twenty or thirty years in the house, (which is not uncommon for lunatics) hence arose the disagreeable necessity of limiting their number, so as not to take more than one half of the paupers of that class, who would occupy the whole house to the exclusion of other cases, which have an equal claim, the Hospital being an asylum not only for maniacs, but all others, except infectious diseases.

Besides ten thousand admitted, it is supposed nearly as many *out-patients* have been attended, from the Dispensary of this institution, and supplied with medicines gratis. These comprehended the poorest classes of people, afflicted with every disease to which the human frame is liable.

At the present time, there are ninety-three patients in the Hospital, of whom sixty-three are on pay, and thirty on the poor list; of the whole number, sixty-three are lunatics.

The buildings are nearly completed, and have cost, as they now stand, about *eighty-four thousand dollars*, towards which, at different times, the Legislature have given *sixty-six thousand dollars*.

Not the least doubt is entertained but they will pay the difference, finish the Hospital, and present it, *a State Contribution*, for the uses intended.

The Hospital exhibits in the centre, a house, sixty-four feet in front, elevated above all the adjoining buildings, and projecting beyond them a proper distance—on the top is a sky-light, to enlighten the theatre for surgical operations; from which there is a beautiful view of the City Plot, the river Germantown, Frankfort, the Fort, and several elegant country seats on the Schuylkill—Two large stair stair cases, leading to the several wards, are made in this division.

Adjoining hereunto on the east, is a ward, eighty feet front, twenty-seven feet deep, and three stories high; at the end, a wing crosses it, north and south, extending in length one hundred and ten feet.

In the middle of the wing, opposite the ward, is a hall, twenty-eight feet square, including a stair case, projecting beyond the other part of the wing, sufficient to cover the cornice, and raised one story above them, with a cupola that affords a secure way out in case of fire.

And adjoining to the centre house on the west, are a ward and wing, similar to those on the east, with this exception, that the wards are about thirty-four feet deep—this extension was agreed to, in order to admit double rows of rooms to accommodate a greater number of lunatics.—The difference, unless to an accurate observer, is scarcely perceivable.

The whole extent of the buildings from east to west, is two hundred and seventy-eight feet; by the length of the wing crossing the wards, the east and west fronts make an agreeable appearance. Detached from the Hospital, at a little distance, is a separate building, with a convenient enclosure for venereal patients, who are kept by themselves.—There are also, sundry other apartments on the lot, such as stable, ice-house, smoke-house, fire engine-house, &c.

Rooms in the Hospital are appropriated to the following uses.

| | | | |
|---|---|---|---|
| For the library | 1 | Steward, matron & maids in the centre house | 8 |
| Contributors | 1 | Lunatics in the west wing and ward | 70 |
| Managers | 1 | Ditto in the east | 16 |
| Museum | 1 | For sick and wounded | 28 |
| Apothecary's shop | 1 | In all | 130 |
| Bathing Rooms | 2 | wards and rooms. | |
| Theatre for operations | 1 | | |
| Wash-house, bake-house and kitchens | 4 | | |
| Cell-keeper and his wife | 1 | | |

The lunatics being separated by the centre house, the latter are not incommoded with their noise. The Capital Stock consists of ground rents, and money at interest, the annual amount of which is about three thousand four hundred dollars; besides this, there is no productive income for for the support of poor patients, except the profit of pay patients, both of which sums united will not maintain more than thirty poor persons ; nor can the number be increased, until by legacies, or future contributions, the funds are enlarged. This is much to be lamented, as every convenience is provided in the Hospital to accommodate three hundred persons on a moderate calculation; but for want of an adequate capital, the contributors are obliged to refuse to numbers, the benefits of an Institution, that is above all others in this part of the world, peculiarly well situated in other respects to relieve them.

The unproductive part of the estate consists in lots of ground bought and paid for by the contributors, and in the Museum and Medical Library.

These lots were mostly purchased early, when land was low, but they are now become valuable, being within the improved parts of the city.

The Hospital stands on a square, three hundred and ninety-six feet in width, and four hundred and sixty-eight feet in length, containing about four acres—round it is a brick wall, and rows of high forest trees. Within the wall, the ground is decorated with gardens, grass plots, gravel walks, hedges, &c.

There is also a vacant square to the east, and one half a square to the west—containing together, more than six acres, running in parallel lines with the ground on which the buildings are erected; the other half of this square is owned by the Alms-house, who mean to keep it always open, so that the Pennsylvania Hospital is situated in the middle of three great squares; which, besides the open streets, measure more than thirteen acres.

The contributors have, also, bought three lots on the south-side of the Hospital.—Their object in providing so much ground was to secure a current of air for the benefit of the sick patients. The policy of this provision was never more conspicuous than during the late fevers, particularly in 1793, when not a person took it in the Hospital, though upwards of four thousand died of it, in about four months, in the city, in that year.

Knowing the inestimable value of open ground to the Hospital, the contributors have a confident assurance, that avarice itself will never dare to propose the alienation of one foot of the ground, which they have provided at their own expence for such a benevolent use.

The Anatomical Museum is a collection of the human body in wax, fine paintings, &c. which may be worth three three thousand dollars.—The paintings are the gift of the late Dr. John Fothergill, of London, valued at one thousand dollars. The other part was the property of the late Dr. Chovet, and purchased of his daughter with a life annuity of fifty pounds sterling per annum. Persons admitted to see this Museum, which is very interesting, pay one dollar each.

The Library comprises about seventeen hundred volumes of choice *Medical Books*, and is thought to be the best collection of the kind in this country; this, and the Museum, are enlarged and supported by a fund of about five hundred dollars per annum, which Medical pupils, who attend the lectures from all parts of the Continent, West Indies, &c. pay for the privilege of reading, and observing the practice of the house; the money is exclusively applied to enlarge the collection, with the consent of the Physicians, who, in other countries, enjoy these perquisites to themselves.

The number who attend the hospital at this season, are about one hundred.

The Managers, Treasurer, and Physicians, are all Contributors, and serve gratis, except that persons in affluence pay the Physicians, as they would if attended in private houses.

Such are the principles on which this institution has been raised and supported—and as it has been of the greatest utility to the public, it is hoped it will continue to excite their attention, until, by the enlargement of its funds, the contributors are enabled to extend the benefits of it to a greater number of poor, agreeably to the design of its pious Founders.

